# The Work Stress Questionnaire (WSQ) – reliability and face validity among male workers

**DOI:** 10.1186/s12889-019-7940-5

**Published:** 2019-11-27

**Authors:** Anna Frantz, Kristina Holmgren

**Affiliations:** 1 Närhälsan Backa Rehabilitation Centre, Rimmaregatan 1C SE-422 55 Hisings Backa, Gothenburg, Sweden; 2 Department of Health and Rehabilitation, Unit of Occupational Therapy, University of Gothenburg, Sahlgrenska Academy, Institute of Neuroscience and Physiology, Box 453, S-405 30 Gothenburg, Sweden

**Keywords:** Questionnaire, Work related stress, Men, Reliability, Face validity, Test retest

## Abstract

**Background:**

The Work Stress Questionnaire (WSQ) was developed as a self-administered questionnaire with the purpose of early identification of individuals at risk of being sick-listed due to work-related stress. It has previously been tested for reliability and face validity among women with satisfying results. The aim of the study was to test reliability and face validity of the Work Stress Questionnaire (WSQ) among male workers.

**Method:**

For testing reliability, a test-retest study was performed where 41 male workers filled out the questionnaire on two occasions at 2 weeks intervals. For evaluating face validity, seven male workers filled out the questionnaire and gave their opinions on the questions, scale steps and how the items corresponded to their perception of stress at work.

**Results:**

The WSQ was, for all but one item, found to be stable over time. The item *Supervisor considers one’s views* showed a systematic disagreement, i.e. there was a change common to the group for this item. Face validity was confirmed by the male pilot group.

**Conclusion:**

Reliability and face validity of the WSQ was found to be satisfying when used on a male population. This indicates that the questionnaire can be used also for a male target group.

## Background

Work-related disorders are a common problem in Sweden as well as in Europe [1, 2]. A survey on work-related disorders that was carried out among workers in Sweden in 2016, found that 26% of the female workforce and 19% of the male experienced disorders related to their working situation [1]. The survey also found that up until 2014, physical conditions have been the predominant cause for work-related disorders among men, however, stress and mental strain has now reached the same levels. Estimating the cost of work-related stress to society is complex, depending on definition of work-related stress and costs associated to it. The cost per year to society has been found to range between 221.3 million USD to 187 billion [3]. The cost for sick-listing in Sweden 2018, taking only into account the cost for rehabilitation benefits and sickness compensation, was approximately 4 billion USD, where stress-related and adjustment disorders represented 20% of all the sick-listing cases [4]. Between the years 2012 and 2016, stress as a cause of work-related disorders in Sweden increased from 6 to 8% for men and from 10 to 15% for women [1]. Along with staggering numbers for sick-leave, a large proportion of the workforce continue to go to work despite experiencing work-related problems [5]. Although there are gender differences in sick-listing, where mental disorders are a more common cause for sick-leave in women [6], sick-listing due to work-related stress is rising among both women and men [1].

It has long been known that several work-related psychosocial factors such as conflicts at work, low influence at work, low co-worker support, poor organizational structure, low justice in interpersonal treatment and decision latitude is connected with sick leave [7–14] and common mental disorders [14, 15]. Interactive effects of poor organizational climate and high work commitment has been found to be associated with a higher rate of sick-leave among both women and men [16]. The world of work is also changing. Boundaries between work and home are challenged when new technology such as smartphones leads to flexible working places and/or hours [17, 18].

Workers tend to experience ill-health due to work-related stress long before sick-listing [9, 19, 20], and often seek help for these complaints at primary health care centers [21]. GPs, however, have reported not having sufficient knowledge on how to address issues related to the patients working situation [22]. Early interventions to address stress-related disorders are of importance [23]. The Work Stress Questionnaire (WSQ) was developed with the intention to identify individuals at risk of sick-leave due to work-related stress. It has been tested for reliability and face validity among women with satisfying results [24]. In the present study, reliability and face validity was to be tested among male workers.

### The questionnaire

The WSQ was developed by Holmgren et al. [24], as a self-administered questionnaire with the purpose of early identification of individuals at risk of being sick-listed due to work-related stress. It consists of only 21 questions, which makes it suitable to use in a clinical setting where time often is sparse. Another advantage is that it is not targeting a specific diagnosis, as other screening tools [25, 26], but can be used to identify work-related stress regardless of the patient’s complaint. The WSQ emanates from the experiences of sick-listed workers and takes into consideration the interaction between personal and environmental factors [24]. It has previously been used in a study to analyze the connection between presence of work-related stress and future work absenteeism in a primary health care setting [9] and a cohort study investigating the association between work-related stress and ill-health/sick-leave in women [27]. In the first study [9], a presence of high stress due to poor organizational climate, especially when coexisting with high personal demands, significantly increased the risk of sick-leave a year later. The women in the cohort study experiencing a higher level of overall work-related stress also had higher rates of self-reported ill-health [27]. Women reporting low influence at work and high stress-levels due to indistinct organization also had a higher probability of sick-leave [27]. In a prospective, longitudinal study the WSQ was found to predict sickness absence for as far as up to 8 years [Knapstad M, Lissner L, Björkelund C, Holmgren K. Organizational climate and work commitment as predictor of 10-year registered sickness absence: The Population Study of Women in Gothenburg, in preparation].

### Objective

Both female and male workers seem to be experiencing ill-health due to work-related stress, and this is often present long before sick-listing [9, 19, 20]. Stress and mental strain as cause of work-related disorders increases, not only among women but also among men [1]. The WSQ was developed using a female reference group (24). Gender differences may influence the psychometric properties of a questionnaire [28], it is therefore important to test reliability and validity of the WSQ for men.

The aim of this study was to evaluate the reliability and face validity of the Work Stress Questionnaire (WSQ) when used on a male working population.

## Method

### Study design

For testing reliability, a test-retest study was performed. The WSQ was filled out by the same respondent on two occasions at 2 weeks intervals. Face validity was evaluated by using a pilot-group that filled out the questionnaire and were encouraged to give comments, either written or oral, concerning the questionnaire. The target group was non-sick-listed employed men aged 18–64 years. The study took place in Gothenburg, Sweden 2017.

### The work stress questionnaire (WSQ)

The WSQ consists of 21 items covering 4 main themes: *Indistinct organization and conflicts*, *Individual demands and commitment*, *Influence at work* and *Work to leisure time interference* (Additional file 1). The questions of the first two themes can be answered *Yes*, *Partly* or *No*. To determine the level of stressfulness in the items of the first two themes, the questions are followed by the question *Do you perceive it as stressful?* The respondent grades the level of stressfulness by answering *Not stressful*, *Less stressful*, *Stressful* or *Very stressful*. The items of the second two themes can be answered *Yes, always*, *Yes, often*, *No, rarely* or *No, never*. Demographic data concerning employment, age and educational level was also collected. In the follow-up questionnaire for the testing of reliability, a question concerning changes at the workplace during the 2-week period was added: *Has anything deviating occurred at your workplace since the first time you filled out the questionnaire that may affect your answers today?* If the respondents answered yes to this question, they were excluded from the study.

### Procedure and data collection

Respondents were recruited from different areas of the labour-market using the researcher’s own social network. Contact persons, who were not involved in any part of the research, were used to identify and approach eligible recruits. The procedure has similarities to snowball sampling [29]. Snowball sampling uses the researchers´ social network to reach a target group with specific characteristics, in this case non-sick-listed employed men. Written information was given to eligible recruits, containing a short background of the study and information about the procedure. Emphasis was laid on the voluntary nature of participation in the study, and the respondents were informed that they could choose to terminate participation at any point without explanation. It was clearly stated in the written information to the participants that consent to participate in the study was given by filling out the WSQ. The completed questionnaire was then put in a sealed envelope and passed on to the research group. This procedure was then repeated 2 weeks later. The questionnaire did not contain any personal information, only a code for matching with the second questionnaire. For this part of the study, a population of 57 employed men, aged 18–64 years, was included. Sixteen of the respondents were excluded, of which 2 respondents did not fill out the second questionnaire and 14 respondents answered yes to the appended question *Has anything deviating occurred at your workplace since the first time you filled out the questionnaire that may affect your answers today?* in the second questionnaire. A total of 41 respondents (*n* = 41) remained for analysis. The demographics of the group are presented in Table 1.

**Table 1:** Characteristics of the participants, *n* = 41

	Men *n* = 41
	% (*n*^a^)
Age	
18–30	19.5 (8)
31–40	43.9 (18)
41–50	26.8 (11)
51–60	4.9 (2)
61–64	4.9 (2)
Educational level	
Primary education	2.4 (1)
Secondary education < 3 years	7.3 (3)
Secondary education > 3 years	19.5 (8)
University or college < 3 years	14.6 (6)
University or college ≥3 years	56.2 (23)
Socioeconomic position	
Higher/intermediate non-manual	67.5 (27)
Lower non-manual	22.5 (9)
Skilled manual	7.5 (3)
Non-skilled manual	2.5 (1)
Hours worked/week	
Full-time	97.6 (40)
Part-time (> 15 h)	2.4 (1)
Employer	
Private	43.9 (18)
Self-employed	4.9 (2)
Public/municipal	36.6 (15)
Public/regional	7.3 (3)
Governmental	2.4 (1)
Other	4.9 (2)

^a^Dispersed numbers are due to internal drop-outs

To evaluate face validity of the WSQ, a pilot-group comprising seven employed men were recruited in the same way as for the test-retest part of the study. The group consisted of men working in both public and private sector, at small and large workplaces and in different positions. The respondents were asked to fill out the WSQ and leave notes, either written or oral, concerning the items and scales. Afterwards, the respondents were encouraged to give comments on scale steps and formulation of the questions as well as if the questionnaire corresponded to their understanding of work-related stress.

### Statistical analysis

**Fig. 1:**
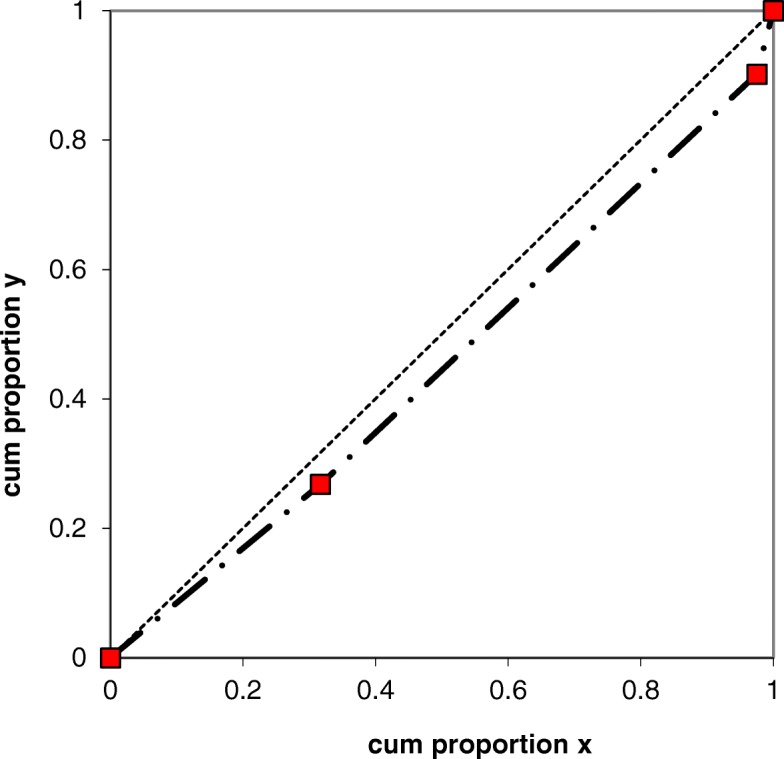
The systematic disagreement of the item *Does your supervisor consider your views?* illustrated by a ROC curve

To analyze the reliability of the questionnaire, a test-retest analysis was performed using a rank-invariant method for analysis of paired ordered categorical data described by Svensson [30]. This method for assessing reliability of a questionnaire has been used previously [24, 31] and is recommended for analysis of ordered categorical data [30]. The method is suitable for analysis of change and is valid regardless of the number of response categories. There is no need for combining or dichotomizing the category distributions. This made it possible to analyze each item of the questionnaire, assessing the occasional and systematic disagreement of each item. As some of the items are divided into two parts, where the first part contains the categories *Yes, Partly, No* and the second part *Not stressful, Less stressful, Stressful, Very stressful,* these two parts were analyzed separately. Percentage agreement was calculated for both parts, the second part was then analyzed further for Relative Rank Variance (RV), Relative Position (RP) and Relative Concentration (RC). For all other items PA, RV, RP and RC were calculated. RV ranges from 0 to 1 and indicates individual changes. The lower the RV-number is, the smaller the occasional disagreement. The items were also analyzed for systematic disagreement by plotting each item in a graph where the x-axis represents the cumulated proportions for the marginal distributions at first test and the y-axis represents the cumulated proportion at retest, see Fig. 1. Each axis ranges from 0 to 1. If there is no disagreement between test and retest, the graph will be plotted as a straight line from point (0, 0) to point [1]. If there is a systematic disagreement, the graph will be either concave or convex. This will be expressed as Relative Position (RP). If there has been a systematic change in concentration, it will be expressed as the Relative Concentration (RC). If there is a change in RC it will result in an S-shaped graph.

Both the RP and RC measurements were calculated for each item. RP and RC ranges from − 1 to 1, where a number close to 0 indicates a low disagreement between test and retest. Both RP and RC refer to changes common to the group. The confidence interval (CI) for RV, RP and RC values for each item was calculated using the bootstrap method, based on the jack-knife standard error. If the CI did not include 0, the item was assessed as having significantly changed between test and retest occasion.

## Results

### Test-retest reliability

The PA of the items ranged from 55 to 98% with a median PA of 77%. To evaluate the stability of the questionnaire, RV, RP and RC were calculated for each item. The result is presented in Table 2. The second parts of two of the items, *Knowledge of work assignments* and *Involved in conflicts at work*, were not analyzed due to a low response rate. The respondent is only requested to answer the second part of these items if they answer the first part with “No” and “Yes” respectively, which explains why the response rate was low for these two items. The first parts of these items were still analyzed for PA. All but one item remained stable over time regarding occasional and systematic disagreement, which means that responses from the two measurements did not vary regarding position on the scale or concentration of responses on group level. RV was close to 0 for all items, implying that individual variation between test and retest was low. However, the confidence interval for RV for the item *Do you take more responsibility at work than you ought to?/stress* was large (RV = 0.14, *CI 0.00–0.39*). The item S*upervisor considers one’s views* showed a significant change in systematic disagreement (RP = 0.10, *CI 0.02–0.18*), shifting towards higher response categories which means grading this item as more stressful on retest occasion. Since the RV value was 0.0 for this item, it cannot be explained by individual variation.

**Table 2:** Values of occasional and systematic disagreement in test-retest study

ITEM			Occasional disagreement		Systematic disagreement			
	*n*	PA	Relative rank Variance (RV)		Relative Position (RP)		Relative Concentration (RC)	
Time to finish assignments	41	83	0.00	(0.00 to 0.01)	0.05	(−0.06 to 0.15)	0.06	(− 0.02 to 0.14)
Influence decisions at work	41	78	0.00	(0.00 to 0.01)	0.01	(−0.10 to 0.11)	−0.02	(− 0.13 to 0.09)
Supervisor consider one’s views	41	88	0.00	(0.00 to 0.00)	**0.10**	**(0.02 to 0.18)**	−0.08	(− 0.17 to 0.02)
Deciding on working pace	41	73	0.01	(0.00 to 0.01)	−0.01	(−0.13 to 0.12)	0.09	(− 0.02 to 0.21)
Workload increased	41	85						
Workload increased/Stress	22	77	0.05	(0.00 to 0.15)	−0.16	(−0.33 to 0.02)	0.04	(− 0.18 to 0.26)
Clear goals at workplace	41	68						
Clear goals at workplace/Stress	16	56	0.10	(0.00 to 0.27)	−0.24	(−0.49 to 0.01)	0.17	(−0.07 to 0.42)
Knowledge of work assignments	41	90						
Knowledge of work assignments/Stress	2^a^							
Clear leadership	41	80						
Clear leadership/Stress	11	55	0.03	(0.00 to 0.09)	−0.02	(−0.31 to 0.26)	0.20	(− 0.03 to 0.43)
Conflicts at work	41	98						
Conflicts at work/Stress	27	67	0.06	(0.00 to 0.18)	−0.13	(−0.30 to 0.03)	0.01	(− 0.20 to 0.22)
Involved in conflicts	29	86						
Involved in conflicts/Stress	6^a^							
Supervisor solved the conflicts	29	69						
Supervisor solved the conflicts /Stress	14	64	0.03	(0.00 to 0.09)	−0.19	(−0.43 to 0.05)	0.05	(−0.40 to 0.50)
High demands	41	90						
High demands /Stress	35	63	0.02	(0.00 to 0.04)	0.02	(−0.12 to 0.17)	0.17	(−0.01 to 0.35)
Engaged	41	98						
Engaged/Stress	38	71	0.06	(0.00 to 0.17)	−0.01	(−0.16 to 0.13)	0.01	(−0.12 to 0.14)
Think about work	41	83						
Think about work/Stress	39	69	0.01	(0.00 to 0.02)	0.02	(−0.11 to 0.15)	0.08	(− 0.06 to 0.23)
Hard to set limits	41	63						
Hard to set limits/Stress	30	67	0.12	(0.00 to 0.31)	−0.06	(−0.24 to 0.12)	0.01	(−0.14 to 0.15)
High responsibility	41	88						
High responsibility/Stress	19	68	0.14	(0.00 to 0.39)	−0.07	(−0.30 to 0.16)	0.11	(−0.13 to 0.34)
Work overtime	41	63						
Work overtime/Stress	26	65	0.02	(0.00 to 0.05)	0.14	(−0.04 to 0.31)	− 0.04	(− 0.20 to 0.12)
Sleep disturbance	41	83						
Sleep disturbance/Stress	16	88	0.00	(0.00 to 0.01)	0.00	(−0.16 to 0.16)	0.00	(−0.06 to 0.06)
Work interfering with family time	41	83	0.00	(0.00 to 0.00)	−0.06	(−0.14 to 0.01)	0.06	(− 0.05 to 0.16)
Work interfering with time to see friends	41	68	0.01	(0.00 to 0.02)	0.00	(−0.11 to 0.11)	−0.02	(− 0.17 to 0.13)
Work interfering with leisure time activities	41	80	0.00	(0.00 to 0.01)	−0.03	(−0.12 to 0.06)	− 0.03	(− 0.15 to 0.10)

Possible RV ranges from 0 to 1 and possible RP and RC ranges from −1 to 1. The 95% confidence interval is in brackets. Significant values of the confidence interval of RV, RP and RC are indicated in bold type

^a^Items are not calculated for RV, RP and RC due to low response rate

### Face validity

Face validity was confirmed by the pilot-group. The participants all confirmed the relevance of the questions regarding work-related stress and the items were found to be generally easy to answer.

## Discussion

For assessing reliability of the questionnaire, a test-retest design was chosen. This design has been suggested as suitable for testing reliability in an already existing questionnaire [28]. The time between test and retest occasion was set to 2 weeks. This interval was chosen so that the respondents probably would have forgotten their responses from the first questionnaire but not too long so that changes in work environment might have occurred [32]. However, ensuring the two occasions to be exactly the same is not possible. To further decrease the possibility of this affecting the answers, the appended question *Has anything deviating occurred at your workplace since the first time you filled out the questionnaire that may affect your answers today?* was formulated. This made it possible to identify respondents who felt that there had been a change in their working conditions and exclude them from analysis.

For statistical analysis a rank-invariant non-parametric method was used, which is suitable for analyzing ordinal data. Pairing of data in this method makes it possible to identify both random individual changes and systematic changes common to the group [30]. Compared to for example weighted Kappa, which is a method commonly used for analyzing changes in data, the rank invariant method is not dependent on the number of categories [33]. Kappa statistics also treat data as nominal. A study comparing the rank-invariant method used in this study and Kappa statistics found the rank-invariant method to be more sensitive detecting changes in data [34].

All but one item showed stability over time. For the item *Supervisor considers one’s views* there was a change common to the group, grading this item as more stressful on retest occasion. The change was however small, with a RP-value of 0.10 (CI 0.02–0.18). How much influence one experiences at work might be something the respondents have not been reflecting on, and may have been made aware of at base-line. At retest they may therefore grade this item with higher response categories. This item was however not commented by the pilot group.

The questionnaire was developed using a female reference group. Gender has been identified as a factor affecting validity of a questionnaire [28], and therefore needs to be evaluated for the target group. A report from the Swedish agency for work environment found that when women and men are exposed to the same stressors at work they respond in a similar way [35]. In a recently published review and meta-analysis, no gender differences were found regarding depressive symptoms when exposed to the same psychosocial work factors related to stress [36]. This supports the findings of this study, that the questionnaire is useful also for a male population. The intent of the questionnaire is to be self-administered and therefore needs to be able to complete without reluctance or hesitation. Few signs of this, for example written comments in the questionnaire or ticking in between scale-pace boxes [37], were found in the test-retest part of the study implying that the attitudes among the participants towards the questionnaire was good.

In the WSQ, four scale-steps are available for determining the level of stressfulness of the items. How many scale-steps that should be used in questionnaires is an issue that is up for debate, where there are both advantages and disadvantages concerning using an even or odd number of scale-steps [37]. Using a scale with four steps forces the respondent to commit him- or herself to either experiencing the item as stressful or not. Since the items remained stable over time, the even number of scale-steps does not seem to affect the reliability of the questionnaire.

The purpose of this study was not to screen for work-related stress but to test the stability of the questionnaire over time. Snowball sampling allowed for recruitment of respondents from the target group.

There are some limitations to the study. In one part of the questionnaire the items are divided into two, where the second part is only answered if the respondent answers positively in the first part of the item. For two of the items, this has resulted in a low response rate to the second part of these items. To increase probability of having enough answers to be able to do a statistical analysis of these items, a larger population sample would have been needed. A larger study population would also have increased the power of the statistical analysis for all items.

Another limitation to the study is that face validity was only confirmed by the pilot group. Face validity may be the weakest form of validation, but has been suggested as a first step in the process of validating a questionnaire [28]. More thorough research to confirm the validity of the questionnaire when used on a male population is however needed.

## Conclusion

An increasing level of men on sick leave due to stress-related disorders calls for a valid instrument for early identification of persons at risk of being put out of work due to these factors. Results from the present study indicate that the WSQ is a reliable and valid questionnaire when used on a male target group. Future research on the development of the questionnaire should focus on more extensive evaluation of validity. The predictive value of the questionnaire on sickness absence in a male population was not in the scope of this article and is also an issue that needs further research.

## Additional file

**Additional file 1.** The Work Stress Questionnaire.

## Data Availability

The datasets used and analyzed during the current study are available from the corresponding author on reasonable request.

## Abbreviations

CI: Confidence interval
PA: Percentage agreement
RC: Relative concentration
RP: Relative position
RV: Relative rank variance
WSQ: Work Stress Questionnaire
