# Supplementary material for: The Work Stress Questionnaire (WSQ) – reliability and face validity among male workers
Source: BMC Public Health. 2019 Nov 27;19:1580. doi: 10.1186/s12889-019-7940-5 (PMC6882173; doi:10.1186/s12889-019-7940-5)
Supplement: Supplementary file 1 — Additional file 1. The Work Stress Questionnaire. [file 12889_2019_7940_MOESM1_ESM.pdf]

The Work Stress Questionnaire

**01** Do you have time to finish your assignments?	□ yes, always<br>□ yes, rather often<br>□ no, seldom<br>□ no, never
**02** Do you have the possibility to influence decisions at work?	□ yes, always<br>□ yes, rather often<br>□ no, seldom<br>□ no, never
**03** Does you supervisor consider your views?	□ yes, always<br>□ yes, rather often<br>□ no, seldom<br>□ no, never
**04** Can you decide on your work pace?	□ yes, always<br>□ yes, rather often<br>□ no, seldom<br>□ no, never
**05a** Has your workload increased?	□ yes<br>□ no – if no: go to question **06a**
**05b** If *yes*: Do you perceive that as stressful?	□ not stressful<br>□ less stressful<br>□ stressful<br>□ very stressful
**06a** Are the goals for your workplace clear?	□ yes – if *yes* continue to question **07a**<br>□ partly<br>□ no
**06b** If *partly* or *no*: Do you perceive that as stressful?	□ not stressful<br>□ less stressful<br>□ stressful<br>□ very stressful
**07a** Do you know which assignments your work tasks include?	□ yes – if *yes* continue to question **08a**<br>□ partly<br>□ no
**07b** If *partly* or *no*: Do you perceive that as stressful?	□ not stressful<br>□ less stressful<br>□ stressful<br>□ very stressful
**08a** Do you know who is making decisions concerning your workplace?	□ yes – if *yes* continue to question **09a**<br>□ partly<br>□ no
**08b** If *partly* or *no*: Do you perceive that as stressful?	□ not stressful<br>□ less stressful<br>□ stressful<br>□ very stressful
**09a** Are there any conflicts at work?	□ yes<br>□ no – if *no* continue to question **12a**
**09b** If *yes*: Do you perceive that as stressful?	□ not stressful<br>□ less stressful<br>□ stressful<br>□ very stressful
**10a** Are you involved in any conflicts at your workplace?	□ yes<br>□ no – if *no* continue to question **11a**
**10b** If *yes*: Do you perceive that as stressful?	□ not stressful<br>□ less stressful<br>□ stressful<br>□ very stressful
**11a** Have your supervisor done anything to solve the conflicts?	□ yes – if *yes* continue to question **12a**<br>□ partly<br>□ no
**11b** If *partly* or *no*: Do you perceive that as stressful?	□ not stressful<br>□ less stressful<br>□ stressful<br>□ very stressful

The Work Stress Questionnaire © Kristina Holmgren, 2008

**12a** Do you put high demands on yourself at work?	□ yes<br>□ no – if *no* continue to question **13a**
**12b** If *yes*: Do you perceive that as stressful?	□ not stressful<br>□ less stressful<br>□ stressful<br>□ very stressful
**13a** Do you often get engaged in your work?	□ yes<br>□ no – if *no* continue to question **14a**
**13b** If *yes*: Do you perceive that as stressful?	□ not stressful<br>□ less stressful<br>□ stressful<br>□ very stressful
**14a** Do you think about work after your working-day?	□ yes<br>□ partly<br>□ no – if *no* continue to question **15a**
**14b** If *yes* or *partly*: Do you perceive that as stressful?	□ not stressful<br>□ less stressful<br>□ stressful<br>□ very stressful
**15a** Do you find it hard to set a limit to work assignment although you have a lot to do?	□ yes<br>□ partly<br>□ no – if *no* continue to question **16a**
**15b** If *yes* or *partly*: Do you perceive that as stressful?	□ not stressful<br>□ less stressful<br>□ stressful<br>□ very stressful
**16a** Do you take more responsibility at work than you ought to?	□ yes<br>□ no – if *no* continue to question **17a**
**16b** If *yes*: Do you perceive that as stressful?	□ not stressful<br>□ less stressful<br>□ stressful<br>□ very stressful
**17a** Do you work after ordinary working hours to finish your assignments?	□ yes<br>□ partly<br>□ no – if *no* continue to question **18a**
**17a** If *yes* or *partly*: Do you perceive that as stressful?	□ not stressful<br>□ less stressful<br>□ stressful<br>□ very stressful
**18a** Do you find it hard to sleep because your mind is occupied with work?	□ yes<br>□ partly<br>□ no – if *no* continue to question **19**
**18b** If *yes* or *partly*: Do you perceive that as stressful?	□ not stressful<br>□ less stressful<br>□ stressful<br>□ very stressful
**19** Due to work, do you find it hard to find time to be with your nearest?	□ yes, always<br>□ yes, rather often<br>□ no, seldom<br>□ no, never
**20** Due to work, do you find it hard to find time to be with your friends?	□ yes, always<br>□ yes, rather often<br>□ no, seldom<br>□ no, never
**21** Due to work, do you find it hard to find time for your recreational activities?	□ yes, always<br>□ yes, rather often<br>□ no, seldom<br>□ no, never
